# Effects of Attention Direction and Perceptual Distraction Within Visual Working Memory

**DOI:** 10.3389/fpsyg.2022.801252

**Published:** 2022-02-21

**Authors:** Weixi Zheng, Liping Jia, Nana Sun, Yu Liu, Jiayang Geng, Dexiang Zhang

**Affiliations:** ^1^Weifang Medical University, Weifang, China; ^2^Luliang University, Lüliang, China

**Keywords:** visual working memory, attention direction, post-stimuli distractor, pre-visual cue, the focus of attention

## Abstract

Although substantial evidence demonstrates that directing attention to specific items is important for improving the performance of visual working memory (VWM), it is still not clear whether the attended items were better protected. The present study, thus, adopted a pre-cueing paradigm to examine the effect of attention direction and perceptual distractor on VWM. The results showed that a valid visual cue improved the individuals’ VWM performances and reduced their reaction time compared to the invalid and neutral cues. However, the VWM performances in the valid and neutral cue conditions were more disrupted by a post-stimuli distractor compared to the invalid cue condition. The findings suggest that although directing attention can improve the VWM performance, it is not efficient in protecting information from being distracted.

## Introduction

Visual working memory (VWM) is a system that temporarily maintains and manipulates visual information to support goal-directed activities (Baddeley, 2012; Baddeley et al., 2019). It is essential in many cognitive activities, such as reading, reasoning, and visual searching; however, its capacity is limited to approximately four items (Luck and Vogel, 2013; Adam et al., 2017). To help the individuals efficiently use this limited capacity and to improve memory performance, researchers often use different methods to direct attention to important memory items (Souza and Oberauer, 2016; Myers et al., 2017; Taylor and Bays, 2018; Oberauer, 2019; Hitch et al., 2020).

The effect of attention direction on VWM has been explored by using a retro-cue paradigm. In this paradigm, a visual cue is presented after the memory array to inform participants that the cued item is most likely to be tested at retrieval. The results have demonstrated that when attention is directed to an item using a retro-cue, this item is memorized with higher accuracy (Griffin and Nobre, 2003; Rerko and Oberauer, 2013; Rerko et al., 2014; Shimi et al., 2014) and better representation precision (Gunseli et al., 2015; Myers et al., 2018; Niklaus et al., 2019). The VWM representation of that item is also better protected from perceptual distraction compared to the uncued items (Makovski and Jiang, 2007; Makovski et al., 2008; Schneider et al., 2017). According to the resource-based model of VWM (Bays and Husain, 2008; Bays and Taylor, 2018), the cueing effect reflects the reallocation of resources toward the cued item from the uncued items, which enhances the quality of the cued item and protects them from distraction.

The effect of attention direction on VWM has also been explored by using a reward paradigm. In this paradigm, the participants were asked to memorize a sequence of items and were informed that one item was more valuable than the others. The results showed that both the final and the more valuable items were better remembered than the other items in the sequence (Hu et al., 2014, 2016; Atkinson et al., 2018a,b; Hitch et al., 2018). However, these items were also more vulnerable to the post-stimuli distractors (Hu et al., 2014, 2016; Hitch et al., 2018). Therefore, the researchers assumed that the final item and the more valuable item were more likely to be stored in the focus of attention, rendering them more accessible; however, these items may also have an increased opportunity to interact with the later on perpetual information, thus being disturbed (Hitch et al., 2020). Allen and Ueno (2018) provided further evidence for the vulnerable characteristic of the valuable items by simultaneously presenting the memory items.

To summarize, although studies using different paradigms have consistently shown that attention improves the VWM performance of the cued items, inconsistencies exist as to whether these items are capable to resist the distractors. Atkinson et al. (2018b) suggested that the reward and cueing manipulations may reflect different mechanisms of attention direction in the VWM. The reward manipulation primarily reflects the function of executive control of the valuable representations, whereas the cueing manipulation mainly reflects attention bias between the memory items. However, in the previous studies, the reward and cueing were manipulated at different stages of the VWM. The cueing manipulations are always at the retention stage of the VWM, whereas the reward manipulations are typically at the encoding stage of VWM. The differences between the effects of different manipulations may reflect that attention has different effects on the different stages of the VWM. The present study, thus, aimed to further reveal how attention direction interacts with the perceptual distractors to influence the VWM by using a pre-cueing paradigm, thereby providing a deeper understanding of the relationship between the different types of attention direction and VWM representation.

## Materials and Methods

### Participants

The required sample size was calculated using a power analysis using G*Power 3.1 (Faul et al., 2009). We predicted a medium effect size (*f* = 0.25) for 80% power at the 0.05 significance level based on *a priori* analysis; the suggested sample size was 19 individuals. To ensure sufficient statistical power and enable comparison with the sample size of previous studies (Allen and Ueno, 2018; Myers et al., 2018), we recruited 24 undergraduate students (female: 12; mean age: 20.67 ± 1.81 years) to participate in the experiment and provided them with monetary compensation. All the participants were recruited from Weifang Medical University and provided written informed consent based on the protocol approved by the Ethics Committee at Weifang Medical University. All the participants were native Chinese speakers, had normal or corrected-to-normal vision, and had no color blindness.

### Equipment and Materials

The experiment was conducted on a Lenovo 19-inch screen with a resolution of 1,024 × 768 pixels at a 60-Hz refresh rate, using E-prime (version 2.0). The memory items were four colored shapes (the visual angle of each item was 1.5° × 1.5°) presented at the corners of an invisible square (3.6° × 3.6°) on a gray background and viewed at approximately 57 cm from the screen. For each trial, the items were chosen at random, without repetition, from a set of 36 items formed by a pool of six colors [red (RGB: 255, 0, 0), yellow (RGB: 255, 255, 0), green (RGB: 0, 255, 0), blue (RGB: 0, 0, 255), purple (RGB: 128, 0, 128), black (RGB: 0, 0, 0)] and six shapes (circle, triangle, cross, moon, square, and ring). The distractor was a flag shape (1.5° × 1.5°) chosen from a set of four colors [pink (RGB: 255, 204, 204), orange (RGB: 224, 128, 62), cyan (RGB: 65, 224, 208), coffee (RGB: 96, 56, 17)], which were different from the colors of the memory and test items. Each of the arrow cues had a visual angle of 1.3° × 1.3°.

### Design and Procedure

The experiment employed a 2 × 3 within-participant design, with cue type (valid, invalid, and neutral) and distractor type (distractor, no-distractor) as factors. In the neutral cue condition, a four-arrow cue was presented before the memory array, each arrow pointed to an item, and the participants were informed that each memory item would be tested 25% of the time. In the valid and invalid cue conditions, an arrow cue was presented before the memory array, and participants were informed that the cued item would be tested 80% of the time (valid condition), while the other 3 items would be tested 20% of the time in total (invalid condition). In the distractor condition, a distractor was inserted at the center of the screen after the memory array; in the no-distractor condition, the distractor was absent.

**FIGURE 1:**
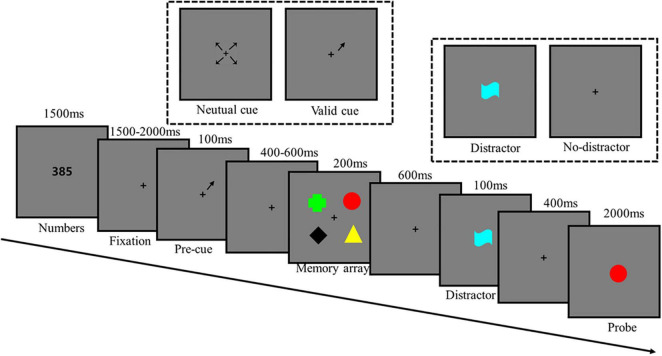
Schematic illustration of the trial procedure.

The experimental procedure is illustrated in Figure 1. Each trial began with a presentation of three numbers for 1,500 ms. Participants were required to repeat the numbers until they were shown the test item to suppress the verbal encoding of the memory stimuli. Then a black fixation cross was presented at the center of the screen throughout the trial. Approximately 1,500–2,000 ms following the onset of the fixation cross, a black arrow serving as a pre-cue was presented for 100 ms. Thereafter, the memory array was presented for 200 ms following a 400–600 ms delay. After a delay of 600 ms, the distractors appeared at the center of the screen for 100 ms, whereas in the no-distractor condition, only the fixation cross appeared. After a 400 ms delay, the probe appeared at the center of the screen for a maximum of 2,000 ms. Participants were required to press specific buttons to indicate whether the probe item was presented in the memory array. They were required to react quickly and accurately. The “yes” and “no” responses were randomly distributed across the blocks (50% chance). The “yes” and “no” responses for the probe item were counter-balanced for each experimental manipulation.

There were 240 trials in total, divided into 4 blocks of 60 trials (each with 40 valid, 10 invalid, and 10 neutral trials) with short rests between the blocks. In each block, half of the trials contained a distractor while the other half did not. The distractor manipulation was counter-balanced between the cue types (valid, invalid, and neutral). The cueing and distractor manipulations were implemented pseudo-randomly across the trials in each block. At the beginning of each block, the participants were informed about the probe probabilities of the items in different cue conditions. At the beginning of the experimental trials, each participant performed 18 practice trials to become familiar with the task. They were then provided feedback regarding their performance on the task only in the practice trials.

### Data Analysis

Reaction times to the probe stimuli and performance accuracy were analyzed using 2 × 3 repeated-measures analysis of variance (ANOVA), with cue type (valid, invalid, and neutral) and distractor type (distractor, no-distractor) as factors. Bonferroni correction was used for multiple comparisons, and *p* < 0.05 was considered significant.

## Results

### Accuracy

**FIGURE 2:**
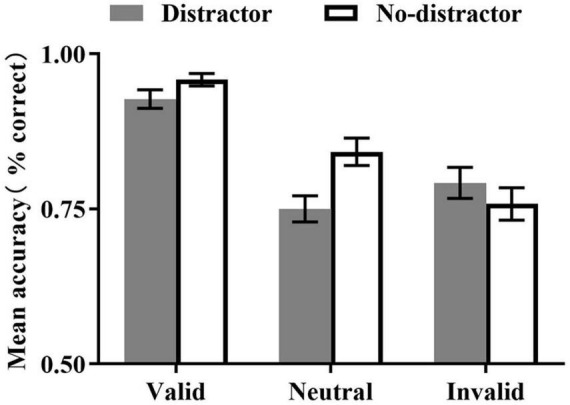
Mean accuracy (±SE) as a function of cue type and distractor.

Mean accuracy is presented in Figure 2. The main effect of the cue type was significant, *F*_(2, 46)_ = 34.46, *p* < 0.001, η*_*p*_*^2^ = 0.60, reflecting higher accuracy in the valid cue condition than in the invalid [*t*(23) = 8.31, *p* < 0.001] and neutral conditions [*t*(23) = 6.47, *p* < 0.001], while no significant difference was observed between the invalid and neutral conditions (*t* = 0.96, *p* = 0.347). The main effect of the distractor type was not significant, *F*_(1, 23)_ = 3.01, *p* = 0.096, η*_*p*_*^2^ = 0.12. The cue type × distractor type interaction was significant, *F*_(2, 46)_ = 5.81, *p* = 0.006, η*_*p*_*^2^ = 0.20. The simple analysis (Multivariate tests) of the interaction showed that accuracy was higher in the no-distractor condition than that of the distractor condition for valid [*F*_(1,__23)_ = 5.18, *p* = 0.032, η^2^*_*p*_* = 0.18] and neutral cue condition [*F*_(1,__23)_ = 8.62, *p* = 0.007, η^2^*_*p*_* = 0.27], but not for invalid cue condition (*F* < 1).

To reveal whether the cueing effects were different in the distractor and no-distractor conditions, we also compared the accuracy between the different cue conditions, respectively, for the different distractor conditions. The results revealed significant pre-cueing effects in both the distractor and no-distractor conditions [distractor condition: *F*_(1,__22)_ = 9.01, *p* < 0.001, η^2^*_*p*_* = 0.45; no-distractor condition: *F*_(1, 22)_ = 50.47, *p* < 0.001, η^2^*_*p*_* = 0.82]. In both the distractor and no-distractor conditions, the accuracy in the valid cue conditions was higher than that of the invalid [distractor condition: *t*(23) = 3.98, *p* < 0.001; no-distractor: *t*(23) = 7.09, *p* < 0.001] and neutral cue [distractor condition: *t*(23) = 3.47, *p* = 0.002; no-distractor: *t*(23) = 6.44, *p* < 0.001] conditions, no differences were observed between the neutral and invalid cue conditions [distractor condition: *t* = 0.96, *p* = 0.347; no-distractor: *t*(23) = 0.28, *p* = 0.780].

### Reaction Times

**FIGURE 3:**
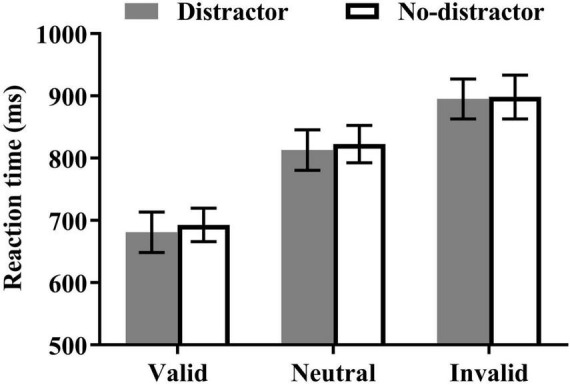
Mean reaction times (±SE) as a function of cue type and distractor.

The mean reaction times are presented in Figure 3. The main effect of the cue type was significant, *F*_(2, 46)_ = 50.43, *p* < 0.001, η*_*p*_*^2^ = 0.69, reflecting a shorter reaction time in the valid cue condition than in the invalid [*t*(23) = −9.35, *p* < 0.001] and neutral conditions [*t*(23) = −7.63, *p* < 0.001], and higher reaction time in the neutral than in the invalid condition [*t*(23) = −3.52, *p* = 0.002]. Neither the main effect of the distractor type nor the cue type × distractor type interaction were significant (*Fs* < 1).

## Discussion

The present study adopted a pre-cue paradigm to examine the effect of attention direction and post-stimuli distractor on the VWM. We replicated the well-established pre-cueing effect (Griffin and Nobre, 2003; Dube et al., 2017; Emrich et al., 2017), with better VWM performances and faster reaction time in the valid cue condition compared to the neutral and invalid cue conditions, indicating that attention direction during VWM encoding facilitates the processing of the cued items. No significant differences were observed in the VWM performances between the invalid and neutral conditions, indicating that the cueing boosts of the cued items were not at the expense of the uncued items. Thus, the visual cue changes how attention is strategically directed across the different items but does not change the VWM capacity or the total attentional resources that are involved in the whole task.

More importantly, we found that the post-stimuli distractors disrupted the VWM performances in the valid and neutral cue conditions but not in the invalid cue condition. However, it is important to note that the VWM performance in the valid cue condition is much better than in the neutral and invalid cue conditions, whether a distractor appeared in the retention interval or not. This indicates that although the cued items are better memorized in the VWM, they are also more disrupted by a post-stimuli distractor, especially compared to the uncued items.

One possible reason for the increased disruption of the items in the valid and neutral cue conditions could be attributed to these items being the focus of attention and thus they possess a greater chance of interaction with the post-stimuli distractors (Hu et al., 2014, 2016; Allen and Ueno, 2018; Hitch et al., 2018). The term focus of attention has been used in two ways. Based on the “broad focus” introduced by the embedded processing model (Cowan, 2001, 2011), the focus of attention can hold approximately four items, which forms the core of working memory and reflects the consciousness of awareness. However, based on the “narrow focus” introduced by the three-embedded-component model (Oberauer, 2002; Oberauer and Hein, 2012), the focus of attention serves to select one item within the working memory for upcoming processing. In the neutral cue condition of the present study, the four memory items had the same possibility to be tested, whereas in the valid cue condition, the cued item had a greater chance to be tested compared to the uncued items. Thus, in the neutral cue condition, all the four items have the same possibility to be stored in the focus, whereas in the valid cue condition, only the cued items can be selected into in the focus. The items either followed a neutral or a valid cue occupy the focus and they have a greater chance to be retrieved. However, when the distractors enters into the focus, they are also more disrupted by these post-stimuli distractors (Hu et al., 2014; Hitch et al., 2018, 2020). It should be noted that numerically (see Tables 1, 2), the disruption effects on the neutral cue conditions was stronger than that of the valid cue condition. That could be because when more items were in the focus, each of them received fewer resources, and their representation was not as accurate and stable as when there only one item was in the focus; thus, disruption effects were stronger in the neutral cue trials than in the valid cue trials. This is consistent with the findings of previous studies using reward paradigm (Allen and Ueno, 2018; Hitch et al., 2018), which have demonstrated that although the focus can hold several items, items are more disrupted when more items are prioritized.

**TABLE 1:** Mean accuracy and standard deviation in each task condition.

Cue type	Distractor	No distractor
Valid cue	0.92 ± 0.06	0.96 ± 0.05
Invalid cue	0.79 ± 0.12	0.76 ± 0.13
Neutral cue	0.74 ± 0.10	0.84 ± 0.11

**TABLE 2:** Mean reaction time and standard deviation (*ms*) in each task condition.

Cue type	Distractor	No distractor
Valid cue	681.14 ± 158.94	692.81 ± 132.13
Invalid cue	895.04 ± 157.19	898.19 ± 173.06
Neutral cue	813.22 ± 159.35	822.53 ± 164.95

Another possibility for the present results could be that the distractor affects the response criterion as opposed to perceptual sensitivity. A comparison of the data on the accuracy and reaction time suggests that there were speed-accuracy trade-offs across distractor-present and distractor-absent trials. On valid trials, the presence of a distractor reduced accuracy (from 96 to 92%) also led to faster responses (reaction time changed from 693 to 681 ms). Numerically, the exact opposite pattern was observed in invalid trials; that is, the accuracy increased while the responses slowed. Thus, we assume that the distractor may affect the response criterion as opposed to perceptual sensitivity. In the present study, the accuracy was near the ceiling (92–96%) in valid trials but at 76–79% in invalid trials. As there was fewer trials in the invalid cue conditions than that of either the valid cue trials, the non-disruption effects in the invalid cue trials maybe reflective of a floor effect. Thus, the performance had a lot of room to drop in the valid trials compared to much less room in the invalid trials.

To summarize, although the suppression of the distractors is of importance to VWM, many studies have already proven how the handling of distractors by the VWM is relatively flexible and diverse (Allon and Luria, 2017, 2019; Makovski, 2019; Mallett and Lewis-Peacock, 2019; Mallett et al., 2020). For example, Allon and Luria (2017) adopted a filtering change-detection task using attention cues to direct irrelevant information and found that attention cues for irrelevant information can help activate filter settings; however, these settings are short-lived. Makovski (2019) used a change-detection task combined with a dot-searching task and found that when the attention system is expecting a distractor, it would be more alert and devote more attention to processing the distractor. The present showed that prioritized items although better remembered were more disrupted by the post-stimuli distractors.

## Conclusion

In conclusion, the present study revealed that pre-visual cues improved the VWM performance of cued items in comparison to the neutral cued and uncued items. Moreover, the cued and neutral cued items were more disrupted by the post-stimuli distractors compared to the uncued items. This could indicate an interaction between the focus of attention in VWM and the perceptual stimulus.

## Data Availability Statement

The raw data supporting the conclusions of this article will be made available by the authors, without undue reservation.

## Ethics Statement

The studies involving human participants were reviewed and approved by the Ethics Committee at Weifang Medical University. The patients/participants provided their written informed consent to participate in this study.

## Author Contributions

WZ wrote the first draft. LJ rewrote the discussion. NS rewrote the data analysis part. YL and JG collected the data and analyzed the data. DZ designed the experiment and rewrote the introduction. All authors took part in the final manuscript.

## Conflict of Interest

The authors declare that the research was conducted in the absence of any commercial or financial relationships that could be construed as a potential conflict of interest.

## Publisher’s Note

All claims expressed in this article are solely those of the authors and do not necessarily represent those of their affiliated organizations, or those of the publisher, the editors and the reviewers. Any product that may be evaluated in this article, or claim that may be made by its manufacturer, is not guaranteed or endorsed by the publisher.
